# A new double-antigen sandwich test based on the light-initiated chemiluminescent assay for detecting anti-hepatitis C virus antibodies with high sensitivity and specificity

**DOI:** 10.3389/fcimb.2023.1222778

**Published:** 2023-11-24

**Authors:** Haicong Li, Shuo Yang, Dan Cao, Qianying Wang, Siyu Zhang, Yi Zhou, Di Liu, Ruifeng Yang, Liyan Cui, Zhaoqin Zhu

**Affiliations:** ^1^ Department of Laboratory Medicine, Shanghai Public Health Clinical Center, Shanghai, China; ^2^ Department of Laboratory Medicine, Peking University Third Hospital, Beijing, China; ^3^ Peking University People’s Hospital, Peking University Hepatology Institute, Beijing, China

**Keywords:** hepatitis C virus, LiCA^®^ anti-HCV, Architect^®^ anti-HCV, double-antigen sandwich, performance evaluation

## Abstract

**Objectives:**

The aim of this study was to evaluate the performance of a new double-antigen sandwich test that is based on the light-initiated chemiluminescent assay (LiCA^®^) for detecting anti-hepatitis C virus antibodies (anti-HCV) in comparison to Architect^®^.

**Methods:**

Analytical characteristics and diagnostic performance were tested using seroconversion panels and large pools of clinical samples. Positive results were validated by the strip immunoblot assay (RIBA) and HCV RNA.

**Results:**

Repeatability and within-lab imprecision of LiCA^®^ anti-HCV were 1.31%–3.27%. The C_5_–C_95_ interval was −5.44%–5.03% away from C_50_. LiCA^®^ detected seroconversion in an average of 28.9 days and showed a mean of 3.7 (*p* = 0.0056) days earlier than Architect^®^. In a pool of 239 samples with known HCV genotypes 1 to 6, both assays correctly detected all subjects. In 16,305 clinical patient sera, LiCA^®^ detected 4 false-negative (0.25‰) and 14 false-positive (0.86‰) anti-HCV cases, while Architect^®^ recorded 6 false-negative (0.37‰) and 138 false-positive (8.46‰) subjects, respectively. Compared to Architect^®^, LiCA^®^ presented a significantly better performance in specificity (99.91% vs. 99.14%, *n* = 16,018, *p* < 0.0001), positive predictive value (95.29% vs. 67.06%, *n* = 419, *p* < 0.0001), and overall accuracy (99.89% vs. 99.12%, *n* = 16,305, *p* < 0.0001), while no significant difference in sensitivity (98.61% vs. 97.91%, *n* = 287, *p* = 0.5217) and negative predictive value (99.98% vs. 99.96%, *n* = 15,886, *p* = 0.3021) was seen. An S/Co value of 3.28 was predicted to be the threshold with a positivity ≥95% for the LiCA^®^ anti-HCV assay.

**Conclusion:**

LiCA^®^ anti-HCV is a precise and fully automatic chemiluminescent assay with superior sensitivity and specificity. The assay can be used as a valuable tool to supplement the diagnosis of HCV infection.

## 1 Introduction

Hepatitis C virus (HCV) causes a heavy burden of liver disease globally. It is estimated that approximately 71 million people have chronic HCV infection and 20% of them might eventually develop into end-stage chronic liver diseases such as liver cirrhosis and hepatocellular carcinoma (World Health Organization, 2017a). Over the last 10 years, HCV-related liver cirrhosis and cancer have increased by 15.5% and 24.8%, respectively (Collaborators GBDCoD, 2017). Although advances in direct-acting antiviral (DAA) therapy regimens enable a high rate (>90%) of cure (Falade-Nwulia et al., 2017; Cooper, 2018), the high burden of undiagnosed infections remains a grand challenge to achieve the goals of the World Health Organization (WHO)—reducing 80% of new HCV infections and 65% of mortality by 2030 (Easterbrook and Group WHOGD, 2016; World Health Organization, 2016). To reach these objectives, at least 90% of infected individuals should be exactly identified and offered timely treatment (Wiktor, 2019).

HCV infection is generally asymptomatic and imperceptible. Laboratory testing plays an essential role for the diagnosis. As recommended by WHO and US Centers for Disease Control and Prevention (CDC), the HCV-antibody (anti-HCV) assay is the initial screening test and the polymerase chain reaction (PCR) test of HCV-RNA is used for confirmation of viremia in reflexing to a reactive anti-HCV test (Centers for Disease Control and Prevention, 2013; World Health Organization, 2017b). Sometimes, a reactive signal does not represent true-positive detection of the antibody due to an unfaithful result of the anti-HCV serological test (Alter et al., 2003). Thereby, the strip immunoblot assay (RIBA) is performed on screening-reactive tests as a supplemental assay with high specificity to confirm the serological antibody status (Alter et al., 2003). Positive results of both antibody and RNA tests indicate an active HCV infection while the antibody-only positive suggests a previously resolved infection (Ghany et al., 2020).

The screening test of anti-HCV is the initial step for the diagnosis of HCV infection. A reliable, highly sensitive and specific, easy-to-use, and affordable kit provides a foundation for access to the WHO targets by 2030, especially in developing countries (Easterbrook and Group WHOGD, 2016). This study is aimed to introduce a new double-antigen sandwich anti-HCV test that is based on the fully automatic homogeneous light-initiated chemiluminescent assay (LiCA^®^), and extensively validate its analytical and clinical performance in comparison to the Architect^®^ anti-HCV assay.

## 2 Materials and methods

### 2.1 Study design

**Figure 1:**
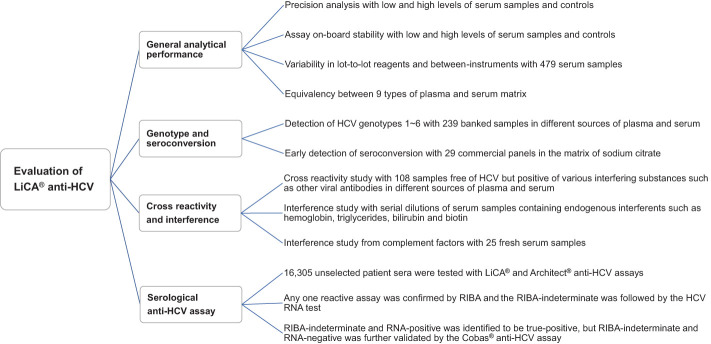
Multicenter performance evaluation of LiCA® anti-HCV. The multicenter evaluation study was conducted across three sites in Beijing and Shanghai, China. The experimental samples for each section were equally assigned to three sites.

We conducted this study to evaluate the analytical and clinical performance of LiCA^®^ anti-HCV across three clinical laboratory centers in Shanghai and Beijing, China. Comprehensive assay specifications including analytical characteristics and diagnostic performance were validated as described in **Figure 1**. All samples were de-identified, separated into vials according to assay frequency and stored at −80°C before testing unless otherwise noted. Tests were performed with samples in freezing once only.

### 2.2 Serological anti-HCV assays

A total of 16,305 unselected clinical patient sera were enrolled for anti-HCV screening tests in parallel on the LiCA^®^ 500 system (Chemclin Diagnostics, Beijing, China) and the Architect^®^ i2000SR system (Abbott Laboratories, IL, USA). LiCA^®^ anti-HCV is a third-generation immunoassay and based on the random-access and fully automatic LiCA^®^ system (Wang et al., 2022). The assay uses two nano-scale beads (streptavidin-coated sensitizer beads and recombinant viral antigen-coated emission beads) and biotin-labeled antigens to bridge a one-step double-antigen sandwich reaction with the biotin-streptavidin amplification format. The antigens contain targeting epitopes from core, NS3, and NS4 domains of the viral protein. After incubation, the immunocomplex can be formed if anti-HCV exists in the sample. The sensitizer and emission beads are linked through the antibody–antigen binding chain, resulting in a short distance (<200 nm) between these two beads. Therefore, the singlet oxygens that are generated from the sensitizer beads can diffuse into the emission beads and initiate a chemiluminescence. When the sample does not contain anti-HCV, there is no binding reaction. A larger distance (>200 nm) between the sensitizer and emission beads blocks the singlet oxygen shifting; thus, no chemiluminescence occurs. With the unique methodology, a LiCA^®^ assay does not need any washing step to separate the immunocomplex from the free components, which may produce interfering signals if not washed out completely during a traditional immunoassay rather than LiCA^®^. Architect^®^ anti-HCV is a two-step indirect immunoassay based on chemiluminescent microparticle technology. The particles contain antigens representing the regions of core, NS3, and NS4. Unlike the LiCA^®^ method, the Architect^®^ assay requires two steps of washing to elute the free components out of the reaction cuvette. Measurement with a ratio of S/Co ≥1.0 is regarded to be reactive and a negative result is considered as S/Co <1.0 for both assays.

**Figure 2:**
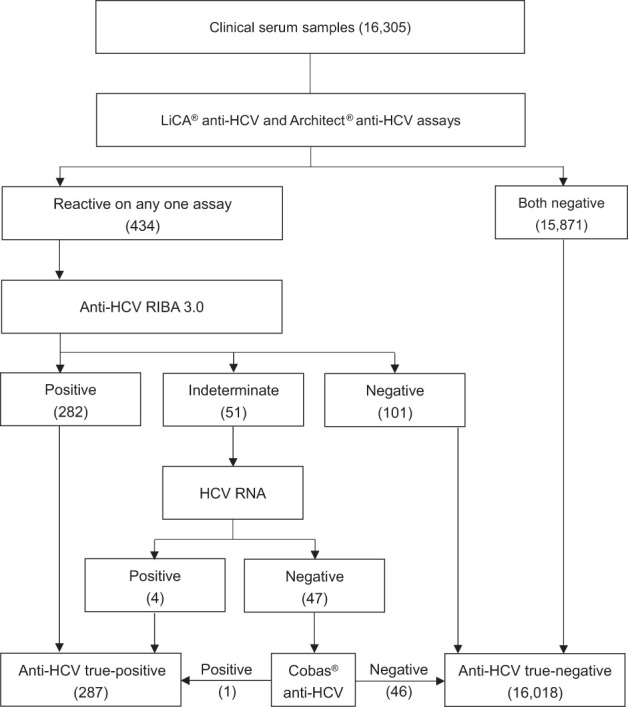
Testing algorithm for the anti-HCV assay.

Subjects with reactive S/Co ratios on any one assay were repeated in duplicate and identified by reflexing with the RIBA 3.0 assay (Mikrogen Diagnostics, Neuried, Germany). The RIBA results were classified as positive, negative, and indeterminate. The RIBA-indeterminate was further investigated using the real-time PCR test (Roche Diagnostics, Mannheim, Germany), which has the limit of detection (LoD) ≤15 IU/mL for HCV RNA. The RIBA-indeterminate but RNA-positive was considered to be true-positive while the RIBA-indeterminate and RNA-negative was further validated with the Roche Cobas^®^ anti-HCV assay. Cobas^®^ anti-HCV is a one-step double-antigen sandwich immunoassay that is based on the electrochemiluminescence method. The result is classified to be reactive as S/Co ≥1.0, non-reactive as S/Co <0.9, or borderline as S/Co within 0.9–1.0. The testing sequence is shown in **Figure 2**.

### 2.3 General analytical characteristics

Precision analysis in S/Co ratios was performed using patient sera and controls following the EP15-A3 protocol of the Clinical and Laboratory Standards Institute (CLSI) (CLSI, 2014). A maximum of 15% in coefficient of variation (CV) was acceptable. The C_50_ test was guided by EP12-A2 using a serial of pooled sera away from both sides of the C_50_ target (CLSI, 2008). Acceptance was considered as the C_5_–C_95_ interval was within C_50_ ± 15%.

Assay on-board stability was evaluated using patient sera and controls refer to EP25-Ed2 (CLSI, 2023). Measurements were consecutively conducted over 20 days. Reloading a new reagent package and re-calibration were not allowed during study of this section. A difference of each point from the mean value of less than 3 times the standard deviation (SD) and 15% was considered to be acceptable.

Variability in lot-to-lot reagents and between-instruments was assessed refer to EP09c (CLSI, 2018) using 479 residual sera that were collected from randomly selected patients with a broad range of S/Co ratios. Comparative data were recorded in parallel. A satisfactory agreement was considered as correlation coefficient *R* > 0.975, slope within 0.85–1.15, intercept close to zero, and bias <15%.

To evaluate plasma equivalency to serum, we prepared 25 groups of anti-HCV-negative samples with 10 different types of matrix in serum and 9 commonly used plasma such as ethylenediaminetetraacetic acid (EDTA), heparin, and citrate. Each group was collected from the same individual free of HCV. Positive samples were prepared with the negative ones by spiking a high-positive anti-HCV specimen at 20:1 volume ratio. S/Co values were recorded for each group at the same time. A satisfactory equivalency was considered as percent difference was <15% in positive samples or S/Co difference <0.15 in negative ones.

### 2.4 Detection of genotypes and seroconversion panels

We used 239 banked samples in different sources of plasma and serum, which has been identified with HCV genotypes by Roche real-time PCR in our labs, and 29 commercially available seroconversion panels in the matrix of sodium citrate for this study. Panels PHV913–926 were from SeraCare (*n* = 6, Milford, MA, USA), HCV6222–10235 were from ZeptoMetrix (*n* = 19, Buffalo, NY, USA), and SCP-HCV-004–012 were from BioMex (*n* = 4, Heidelberg, Germany). All samples were measured on LiCA^®^ and Architect^®^ anti-HCV in parallel.

### 2.5 Cross-reactivity and interference

We collected 108 samples free of HCV but positive of interfering substances, such as other viral antibodies, human anti-mouse antibody (HAMA), and auto-antibodies, to evaluate cross-reactivity from potential interferents. The specimens included different sources of plasma and serum samples. All samples were measured on LiCA^®^ and Architect^®^ in parallel. A significant cross-reactivity was considered as a reactive S/Co was observed in a negative sample unexpectedly.

To identify possible interferences from hemoglobin, triglycerides, bilirubin, and biotin, we used two patient sera containing anti-HCV of 0.71 and 4.75 S/Co, respectively. Patient samples were diluted with serial concentrations of each interferent. S/Co values were recorded on each dilution. A significant interference was considered as percent recovery change ≥15%.

Complement factors are mainly present in fresh blood samples and disappear rapidly during storage. To rule out the potential influence of complement factors on the assay, 25 fresh HCV-negative sera were spiked with a fresh serum sample in strong-positive to anti-HCV to obtain 5 low- and 20 high-positive fresh pools. All samples were stored at 2–8°C and replicate tests were performed for each dilution on days 0, 1, 2, 3, and 4. A significant influence was considered as assay difference from the baseline S/Co on day 0 was ≥15%.

### 2.6 Statistics

All data were analyzed by the software program MedCalc 20.0.22 (MedCalc Software, Mariakerke, Belgium) and Excel 2019 (Microsoft, WA, USA). Anti-HCV screening tests were classified to be reactive as S/Co ≥1.0 or nonreactive as S/Co <1.0. Positive and negative results were confirmed upon RIBA and HCV RNA assays. A negative–positive reverse conversion was regarded to be primarily significant from the qualitative perspective. More valuable information was obtained with quantitative analysis in S/Co ratios. A statistical significance was considered as *p* < 0.05.

## 3 Results

### 3.1 General analytical characteristics

Based on the assay S/Co values, repeatability and within-lab imprecision of LiCA^®^ anti-HCV were determined to be 1.31%–3.27% across low and high levels of quality controls (QC) and patient sera (**Supplemental Table 1**). From the perspective of qualitative analysis, the C_5_–C_95_ interval was identified to be −5.44%–5.03% away from the C_50_ target (**Supplementary Figure 1**). LiCA^®^ anti-HCV presented an excellent analytical precision.

**Figure 3:**
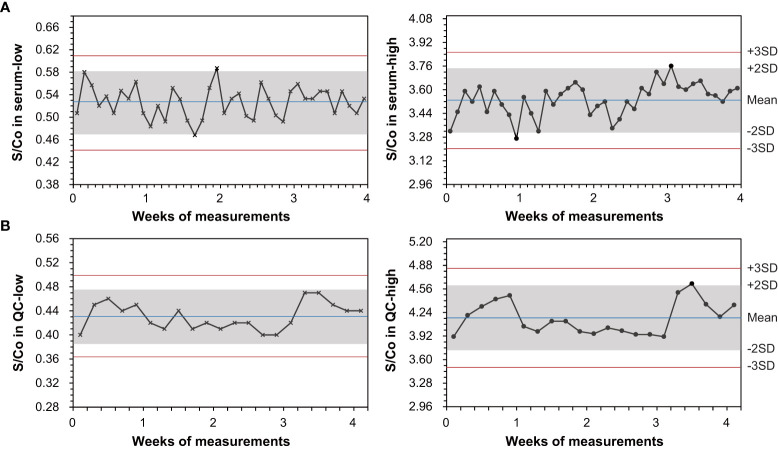
Evaluation of on-board stability for the LiCA® anti-HCV assay following the EP25-Ed2 protocol. Measurements were performed consecutively over 20 days, using low and high signal-to-cutoff (S/Co) ratios of pooled-serum specimens and quality controls (QC), respectively. A reactive S/Co is showing in the symbol (•) and a non-reactive S/Co is in the symbol (×).

**Figure 3** shows daily waves of the assay S/Co on pooled sera and controls over a consecutive 20-day monitoring and demonstrates a good on-board stability. Most measurements were narrowed in a deviation within ±2 SD from the average. Only few points jumped slightly over the line of ±2 SD. In low and high S/Co levels of serum samples, total CV was 5.21% and 3.09%, respectively. The assay difference of each point from mean was within −11.18%–11.40%. In controls, total CV was 5.22% in QC-low and 5.36% in QC-high, respectively. The assay difference of each point from mean was within −7.08%–11.32%. No negative–positive reverse conversion was observed during 4 weeks of assays.

A good concordance was validated between different reagent lots and analyzers in serum pools with either full range (0.05–380) or low levels (<10) of S/Co ratios (**Supplementary Figure 2**). The Spearman correlation coefficient was determined to be 0.976–0.998 and mean bias was 0.33%–4.94%. At the reactive cutoff value (S/Co =1. 0), assay difference from lot-to-lot reagents and between-instruments was estimated to be −1.32%–1.27%.

No significant deviation was observed in nine types of commonly used plasma from serum matrix (**Supplemental Table 2**). In negative samples, assay S/Co values in any kind of plasma were measured to be almost equivalent to those in serum from the same individual. In positive groups, we recorded a minus but acceptable bias (−11.26 to −3.15%) in plasma from serum. No reverse conversion between negative and positive expectations was observed.

### 3.2 Detection of seroconversion panels and HCV genotypes

Early detection of HCV infection was evaluated with commercially available seroconversion panels in sodium citrate (**Table 1**). Of 29 panels studied in total, LiCA^®^ presented 14 in earlier (mean −8.8 days, range −28 to −3 days), 3 in later (mean +5.3 days, range 3–11 days), and 12 in equal detection compared to Architect^®^. Overall, LiCA^®^ detected seroconversion in an average of 28.9 (95% CI: confidence interval, 18.4–39.3) days and showed mean 3.7 (6.4–1.0) days earlier than Architect^®^. The Wilcoxon test demonstrated that there was a significant difference in seroconversion detection between two assays (*p* = 0.0056).

**Table 1:** Detection of seroconversion panels in the sample matrix of sodium citrate.

Seroconversion panel (*n* = 29)	Days since the first reactive bleed			Numbers of reactive bleeds/total		
	LiCA^®^ anti-HCV	Architect^®^ anti-HCV	LiCA^®^ vs. Architect^®^	LiCA^®^ anti-HCV	Architect^®^ anti-HCV	LiCA^®^ vs. Architect^®^
PHV913	9	7	2	1/4	2/4	1
PHV915	5	12	−7	3/4	2/4	−1
PHV919	0	28	−28	7/7	3/7	−4
PHV920	7	13	−6	9/10	8/10	−1
PHV925	10	27	−17	2/5	1/5	−1
PHV926	0	14	−14	5/5	1/5	−4
HCV6222	36	40	−4	2/8	1/8	−1
HCV6224	11	19	−8	3/6	2/6	−1
HCV6225	73	78	−5	3/16	2/16	−1
HCV6226	32	37	−5	5/12	4/12	−1
HCV6227	74	74	0	2/7	2/7	0
HCV6228	28	31	−3	4/12	3/12	−1
HCV6229	17	17	0	4/8	4/8	0
HCV9041	62	62	0	4/8	4/8	0
HCV9044	21	21	0	3/6	3/6	0
HCV9045	37	37	0	2/8	2/8	0
HCV9047	28	28	0	4/10	4/10	0
HCV9050	86	83	3	1/14	3/14	2
HCV9054	82	82	0	1/10	1/10	0
HCV9058	7	10	−3	3/5	2/5	−1
HCV9094	0	7	−7	5/5	3/5	−2
HCV9095	21	10	11	2/5	3/5	1
HCV10071	77	77	0	5/7	5/7	0
HCV10165	24	24	0	4/9	4/9	0
HCV10235	6	6	0	3/5	3/5	0
SCP-HCV-004	10	10	0	2/5	2/5	0
SCP-HCV-009	52	52	0	3/8	3/8	0
SCP-HCV-010	15	25	−10	5/9	2/9	−3
SCP-HCV-012	7	13	−6	3/5	1/5	−2
Total	837	944	−107	100/223	80/223	−20
Mean (95% CI)	28.9 (18.4 to 39.3)	32.6 (22.9 to 42.2)	−3.7 (−6.4 to −1.0)	N/A^a^	N/A	−0.7 (−1.2 to −0.2)

a N/A, not applicable.

A total of 239 banked samples were used for detection of HCV genotypes (**Supplemental Table 3**). Both LiCA^®^ and Architect^®^ correctly detected all samples with reactive S/Co values.

### 3.3 Assay specificity to potential interferents

No significant cross-reactivity was detected on LiCA^®^ to 19 sources of potential interferents such as various viral antibodies, HAMA, and auto-antibodies (**Supplemental Table 4**). Only background signals (S/Co < 0.2) were recorded in all 108 samples free of HCV but positive of interfering substances.

A percent recovery change <15% was observed in both low and high S/Co levels of specimens (0.71 and 4.75) by spiking interferents up to 169.5 mmol/L triglycerides, 4,276.0 µmol/L bilirubin, 5.0 g/L hemoglobin, or 409.0 nmol/L biotin, respectively.

The assay difference on day 1–4 storage from the baseline S/Co on day 0 was determined to be −1.37%–8.85% and −2.65%–10.34% in 5 low-positive (mean S/Co = 5.80) and 20 high-positive (mean S/Co = 84.10) fresh serum pools, respectively. No significant influence was observed on the assay to complement factors.

### 3.4 Diagnostic performance in clinical samples

Of 16,305 clinical patient serum samples, 287 were confirmed to be anti-HCV true-positive and 16,018 were true-negative (**Figure 2**). The diagnostic performance (95% CI) for anti-HCV detection is summarized in **Table 2**. Compared to Architect^®^, LiCA^®^ presented a significantly better performance in specificity (99.91% vs. 99.14%, *n* = 16,018, *p* < 0.0001), positive predictive value (PPV, 95.29% vs. 67.06%, *n* = 419, *p* < 0.0001), and overall accuracy (99.89% vs. 99.12%, *n* = 16,305, *p* < 0.0001), while no significant difference in sensitivity (98.61% vs. 97.91%, *n* = 287, *p* = 0.5217) and negative predictive value (NPV, 99.98% vs. 99.96%, *n* = 15,886, *p* = 0.3021). Among 16,305 patient sera, LiCA^®^ detected 4 false-negative (0.25‰) and 14 false-positive (0.86‰) anti-HCV cases, while Architect^®^ recorded 6 false-negative (0.37‰) and 138 false-positive (8.46‰) subjects, respectively (**Table 3**). We listed all of 10 false-negative assays in terms of S/Co, positive RIBA bands, and RNA results in **Table 4**. LiCA^®^ presented 100% of agreement with Cobas^®^. There was one case that was confirmed to be positive (857 IU/mL) by HCV RNA. LiCA^®^ detected with a good-reactive S/Co of 12.11 but Architect^®^ misdiagnosed with an entirely nonreactive S/Co of 0.05.

**Table 2:** Diagnostic performance (95% CI) of the assays in clinical patient serum samples.

*n* = 16,305	LiCA^®^ anti-HCV	Architect^®^ anti-HCV	*p*-value
Sensitivity, %	98.61% (96.47%–99.62%)	97.91% (95.51%–99.23%)	0.5217
Specificity, %	99.91% (99.85%–99.95%)	99.14% (98.98%–99.28%)	<0.0001
Negative predictive value, %	99.98% (99.93%–99.99%)	99.96% (99.92%–99.98%)	0.3021
Positive predictive value, %	95.29% (92.29%–97.15%)	67.06% (63.28%–70.64%)	<0.0001
Accuracy, %	99.89% (99.83%–99.94%)	99.12% (98.96%–99.26%)	<0.0001

**Table 3:** Performance of the assays stratified by signal-to-cutoff (S/Co) ratios in clinical patient serum samples.

Assay S/Co^a^ stratification	No. (%) of LiCA^®^ anti-HCV in each group			No. (%) of Architect^®^ anti-HCV in each group			LiCA^®^ vs. Architect^®^ agreement (95% CI)
	Subjects	False-negative or positive	Negative or false-positive	Subjects	False-negative or positive	Negative or false-positive	
<1.00 (nonreactive)	16,008	4 (0.02%)	16,004 (99.98%)	15,886	6 (0.04%)	15,880 (99.96%)	99.91% (99.84%–99.95%)
<0.90	16,007	4 (0.02%)	16,004 (99.98%)	15,877	5 (0.03%)	15,872 (99.97%)	99.91% (99.85%–99.95%)
0.90–0.99	1	0	1 (100.0%)	9	1 (11.11%)	8 (88.89%)	88.89% (51.75%–99.72%)
≥1.00 (reactive)	297	283 (95.29%)	14 (4.71%)	419	281 (67.06%)	138 (32.94%)	67.30% (62.58%–71.78%)
1.00–2.99	21	7 (33.33%)	14 (66.67%)	137	23 (16.79%)	114 (83.21%)	18.98% (12.79%–26.56%)
3.00–4.99	4	4 (100.0%)	0 (0.00%)	28	14 (50.00%)	14 (50.00%)	50.00% (30.65%–69.35%)
≥5.00	272	272 (100.0%)	0 (0.00%)	254	244 (96.06%)	10 (3.94%)	95.28% (91.89%–97.54%)
Total	16,305	287 (1.76%)	16,018 (98.24%)	16,305	287 (1.76%)	16,018 (98.24%)	99.07% (98.91%–99.21%)

a S/Co, signal-to-cutoff ratio.

**Table 4:** Overview of false-negative assays on LiCA^®^ and Architect^®^ anti-HCV in a cohort of 16,305 patient sera (*n* = 10).

Architect^®^ S/Co^a^	LiCA^®^ S/Co^a^	Cobas^®^ S/Co^a^	RIBA 3.0	Positive bands on RIBA	HCV RNA^b^	Anti-HCV confirmatory
7.98	0.06	0.07	Positive	Core2, NS3	Negative	Positive
5.84	0.09	0.10	Positive	Core1, NS3	Negative	Positive
1.55	0.07	0.04	Positive	Core2, NS3, Helicase	Negative	Positive
1.36	0.10	0.04	Positive	NS3, NS4, NS5, Helicase	Negative	Positive
0.27	3.07	14.33	Positive	Core1, Core2, NS3, NS4	Negative	Positive
0.17	1.70	12.19	Positive	Core1, Core2, NS3, Helicase	Negative	Positive
0.18	1.38	11.23	Positive	Core1, Core2, NS3, NS5	Negative	Positive
0.12	1.20	6.42	Positive	Core1, Core2, NS3	Negative	Positive
0.94	54.73	59.66	Indeterminate	NS3, NS4	Negative	Positive
0.05	12.11	26.17	Indeterminate	NS3, NS4	857 IU/mL	Positive

a Measurement with a ratio of signal-to-cutoff (S/Co) ≥1.0 was regarded to be reactive and a negative result was considered as S/Co <1.0 for both LiCA^®^ and Architect^®^ assays. For the Cobas^®^ assay, S/Co ≥1.0 was reactive and S/Co <0.9 was non-reactive, while S/Co between 0.9 and 1.0 was classified to be borderline.

b The limit of detection (LoD) of the HCV RNA test was ≤15 IU/mL.

**Figure 4:**
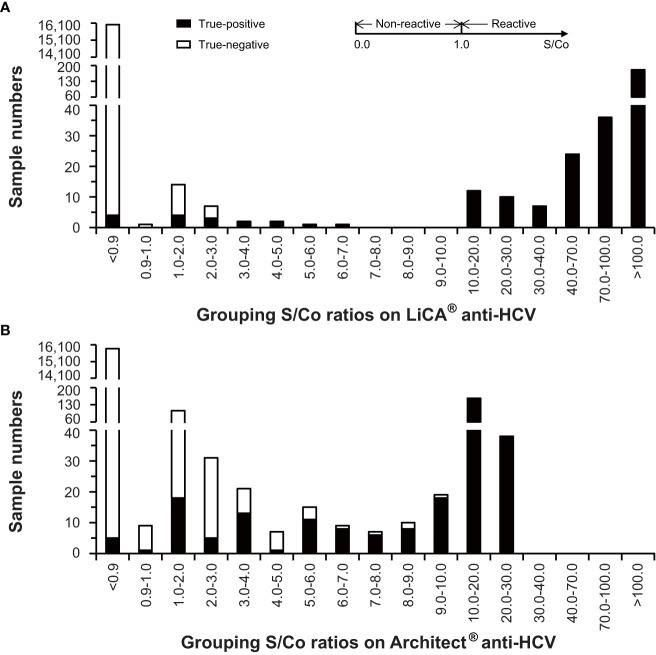
Distribution of signal-to-cutoff (S/Co) ratios on LiCA^®^ and Architect^®^ anti-HCV assays in clinical patient sera (n=16,305).

With stratified S/Co ratios in a range of 1.00–2.99, 3.00–4.99, and ≥5.00, and overall tested-reactive results (≥1.00), the proportion of confirmatory positive results was determined to be 33.33% (*n* = 21), 100% (*n* = 4), and 100% (*n* = 272), and 95.29% (*n* = 297) on LiCA^®^ in contrast to 16.79% (*n* = 137), 50.00% (*n* = 28), and 96.06% (*n* = 254), and 67.06% (*n* = 419) on Architect^®^, respectively (**Table 3**). The distribution of S/Co ratios in more detailed stratification clearly revealed that Architect^®^ had much more false-positive tests especially in a range of 1.0–5.0 S/Co (**Figure 4**). LiCA^®^ reported the results with much wider distribution of S/Co ratios (0–475.87 vs. 0–24.94) and less count in the “borderline” range of 0.9–5.0 S/Co (26 vs. 174) than Architect^®^ did.

Probit regression estimated that the predicting S/Co value (95% CI) with a positivity ≥95% was 3.28 (2.74–4.12) on LiCA^®^ (**Supplementary Figure 3**). Using the predicting value as a cutoff, the data set of this study presented 100% of PPV and 99.93% of NPV.

### 3.5 Discrepancy between LiCA^®^ and Architect^®^ in clinical samples

LiCA^®^ showed a weak correlation with Architect^®^ based on the assays with S/Co ≥1.0 (**Supplementary Figure 4A**, *R* = 0.296, *p* < 0.0001). An improved correlation was observed with logarithmic transformation of S/Co ratios (**Supplementary Figure 4B**, *R* = 0.650, *p* < 0.0001). Overall agreement (95% CI) between both methods was 99.07% (98.91%–99.21%, *n* = 16,305) with Cohen’s kappa 0.78 (0.75–0.82). Positive and negative agreements were 67.30% (62.58%–71.78%, *n* = 419) and 99.91% (99.84%–99.95%, *n* = 15,886), respectively (**Table 3**).

A total of 152 (0.93%, *n* = 16,305) discordant measurements were observed between these two assays (**Supplemental Table 5**). Most discrepancies (133/152, 87.50%) resulted from the false-positive assays on Architect^®^, especially from those with a lower reactive S/Co between 1.0 and 5.0 (123/133, 92.48%). For these discordant assays between LiCA^®^ and Architect^®^, LiCA^®^ presented a high agreement with Cobas^®^ (143/152, 94.08%). There were 47 (0.28%, *n* = 16,305) discordances with RIBA-indeterminate but RNA-negative results. The Cobas^®^ anti-HCV assay was used for further validation and confirmed that 46/47 were false-positive and 1/47 was false-negative from Architect^®^. A perfect consistency (47/47, 100%) was observed between LiCA^®^ and Cobas^®^ assays. The detailed records were summarized in **Supplemental Table 6**.

## 4 Discussion

Typically, HCV RNA can be detected in blood within 1–3 weeks while the antibody seropositivity occurs from several weeks to months after viral exposure (Cloherty et al., 2016; Ghany et al., 2020). Among patients with acute HCV infection, approximately 50%–80% individuals become chronic cases while 20%–50% of the subjects achieve spontaneous clearance (Kamal, 2008). In general, a positive HCV RNA test confirms an early infection during the “window” stage of seroconversion and the viremia status of a current infection (acute and chronic) while the anti-HCV-positive indicates a prior resolved infection or a current virus carrier. During the early stage of infection, people may develop different levels of antibodies due to varied immunogenicity of HCV antigens and different reactivity of individuals. The detection capability for a given anti-HCV assay is highly associated with the antigen configuration of the reagent kit because each HCV antigen may have unequal contribution to antibody detection (Warkad et al., 2019; Jiang et al., 2021). Both WHO and US CDC guidelines on HCV tests recommend that the diagnosis of infection is initially detected with a serological screening of anti-HCV and followed by a reflexive PCR test of HCV RNA (Centers for Disease Control and Prevention, 2013; World Health Organization, 2017b). A probable false-negative anti-HCV test due to the window period (World Health Organization, 2017b) or delayed seroconversion (Thomson et al., 2009) may lead to a misdiagnosis while too many false-positive screening detections would cause high burden of expensive RNA assays and unnecessary medical visits (Alter et al., 2003; Centers for Disease Control and Prevention, 2013). Thereby, a reliable anti-HCV screening kit with high sensitivity and specificity is essential (Easterbrook and Group WHOGD, 2016).

In this study, LiCA^®^ demonstrated superior sensitivity to Architect^®^ for the early detection of HCV infection. Of the 29 seroconversion panels studied in total, LiCA^®^ detected 14 in earlier (mean −8.8 days) and only 3 in later (mean +5.3 days) detection than Architect^®^ did. LiCA^®^ presented a significantly shorter window phase (overall −3.7 days, *p* = 0.0056) in comparison to Architect^®^. LiCA^®^ also correctly detected all subjects in a pool of 239 samples with known HCV genotype 1–6. Using a large pool of clinical patient sera (*n* = 16,305), LiCA^®^ showed a slightly better performance in diagnostic sensitivity (98.61% vs. 97.91%) compared to Architect^®^. Notably, LiCA^®^ and Architect^®^ respectively detected four and five false-negative cases that were confirmed to be anti-HCV-positive but RNA-negative. Undetectable HCV RNA may indicate a prior resolved infection in which the virus has been eradicated from the circulation (El Ekiaby et al., 2015). Inconsistency of detection performance in the same cohort may be explained by the specific antigen configuration of different anti-HCV assays (Warkad et al., 2019; Jiang et al., 2021). An additional one that was misdiagnosed on Architect^®^ with an entirely nonreactive S/Co (0.05) was well-reactive (12.11) on LiCA^®^ and confirmed to be positive (857 IU/mL) by HCV RNA. The indeterminate RIBA test showed positive bands to antigens representing NS3 and NS4 regions. This case might be associated with the early stage of an acute infection. It has been demonstrated that high rates of the viral transmission occurred at all levels of viremia, whether anti-HCV was positive or negative (Operskalski et al., 2003). Factors for misdiagnosis on Architect^®^ could be less sensitivity in detecting antibodies to NS3 and NS4 proteins (Myrmel et al., 2005) and lack of capture to anti-HCV IgM on an indirect immunoassay (Wu et al., 2008).

Among of 16,305 clinical samples enrolled, the comparative Architect^®^ assay recorded 138 false-positive results. In low levels of S/Co ratios (1.0–5.0) on Architect^®^, over three-fourths of assays were exclusive of true positivity. Compared to Architect^®^, LiCA^®^ detected only 14 false-positive cases in the same cohort and presented a significantly better specificity (99.91% vs. 99.14%, *n* = 16,018, *p* < 0.0001) and PPV (95.29% vs. 67.06%, *n* = 419, *p* < 0.0001). Cross-reactivity and interfering experiments also demonstrated that LiCA^®^ anti-HCV did not show any response to 19 sources of potential interferents studied. The exceptionally high specificity plus high sensitivity enables LiCA^®^ to deliver a more accurate diagnosis, in which negative and positive cases can be better discriminated with a wider distribution of S/Co but with a much lower count in the “borderline” (S/Co 0.9–5.0) range (**Figure 4**), thus facilitating a timely treatment. Using the threshold of 3.28 S/Co that was determined herein to predict positivity ≥95% may further improve reflexive testing sequence for HCV infection (Lai et al., 2011; Choi et al., 2018).

The nature of high sensitivity and specificity on LiCA^®^ anti-HCV can be attributed to the assay methodology. LiCA^®^ anti-HCV is based on the double-antigen sandwich format. Two levels of antigen specifically clamp the antibody to reduce nonspecific binding minimally. In the indirect method that is used on Architect^®^, however, the labeled second anti-IgG antibodies can recognize not only the targeting antibodies but all human IgG molecules, thus detecting signals in combination of nonspecific bindings and giving false-positive results (Wu et al., 2008). Previous studies confirmed a high rate of false-positive detection on Architect^®^ anti-HCV especially in low S/Co (Ha et al., 2019; Yang et al., 2019; Lucey et al., 2022). A two-screening-test strategy was proposed to relieve the high burden of further confirmatory tests because a false-positive result was rarely seen on two different serological assays (Vermeersch et al., 2008; Easterbrook and Group WHOGD, 2016). However, ruling out the discordant reactive anti-HCV by repeating tests may yield more false-negative diagnoses (Parry et al., 2017). Hence a reliable screening test with high sensitivity and specificity is critical. Interestingly, a high agreement (94.08%) between LiCA^®^ and Cobas^®^ was observed in the cohort of 152 discrepancies between LiCA^®^ and Architect^®^. Sharing a similar methodology in a double-antigen sandwich format on both LiCA^®^ and Cobas^®^ might explain this. Generally, sample dilution is used to mitigate interference from nonspecific IgG captures in the indirect format. In contrast, the sandwich method allows assay in an undiluted specimen, consequently offering better detection despite the low levels of antibodies. Another advantage ascribed to the sandwich format is the enhanced detectability to anti-HCV IgM that is from a newly infected individual but cannot be recognized in the indirect assay (Wu et al., 2008). Improved sensitivity on LiCA^®^ can also be obtained from the bigger specific surface area on the nano-scale particles and from signal amplification on the biotin-streptavidin mechanism (Wu et al., 2008; Yu et al., 2023). Recent findings have validated the superior assay capability of the LiCA^®^ method in detecting thyrotropin, cardiac troponin, and severe acute respiratory syndrome coronavirus type-2 (SARS-CoV-2) antigen (Wang et al., 2022; Yang et al., 2022; Yu et al., 2023).

Along with high sensitivity and specificity, LiCA^®^ anti-HCV was characteristic of excellent analytical precision and stability in day-to-day, lot-to-lot, different instruments, and sample matrix. Moreover, the assay was performed on a no-wash homogeneous immunoassay platform with a fully automatic walkaway model and gained advantages in flexibility, productivity, and cost-effectiveness to adapt varied testing demand towards ending HCV worldwide.

The limitation of this study is incomplete RNA testing data and missing medical history and patient follow-up. Although discriminating the true antibody-positive from the false-positive by RIBA is informative of infection status (Rafik et al., 2016), a further investigation of the correlation of HCV-antibody titer to active viremia is necessary.

In conclusion, LiCA^®^ anti-HCV is a precise and fully automatic chemiluminescent assay with superior sensitivity and specificity. The assay can be used as a valuable tool to supplement the diagnosis of HCV infection.

## Data availability statement

The original contributions presented in the study are included in the article/**Supplementary Material**. Further inquiries can be directed to the corresponding authors.

## Ethics statement

Research involving human subjects complied with all relevantnational regulation and institutional policies and is in accordancewith the tenets of the Helsinki Declaration (as revised in 2013), and has been approved by the Ethics Committee of Shanghai PublicHealth Clinical Center (2018-E091-01).

## Author contributions

All authors listed have made a substantial, direct, and intellectual contribution to the work, and approved it for publication.
