# Supplementary material for: A new double-antigen sandwich test based on the light-initiated chemiluminescent assay for detecting anti-hepatitis C virus antibodies with high sensitivity and specificity
Source: Front Cell Infect Microbiol. 2023 Nov 24;13:1222778. doi: 10.3389/fcimb.2023.1222778 (PMC10704264; doi:10.3389/fcimb.2023.1222778)
Supplement: Supplementary file 1 [file Table_1.docx]

**Supplemental Table 1:** Precision analysis of the LiCA^®^ anti-HCV assay in serum samples and controls.

| Sample | Mean |  | Repeatability | |  | Within-lab imprecision | |
| --- | --- | --- | --- | --- | --- | --- | --- |
|  | (S/Co^a^) |  | SD | CV% |  | SD | CV% |
| Analyzer 1^#^ | | | | | | | |
| QC L | 0.40 |  | 0.010 | 2.44 |  | 0.012 | 2.95 |
| QC M | 3.98 |  | 0.056 | 1.42 |  | 0.073 | 1.84 |
| QC H | 15.79 |  | 0.322 | 2.04 |  | 0.413 | 2.62 |
| Serum L | 1.05 |  | 0.019 | 1.80 |  | 0.023 | 2.16 |
| Serum H | 14.80 |  | 0.194 | 1.31 |  | 0.261 | 1.76 |
| Analyzer 2^#^ | | | | | | | |
| QC L | 0.41 |  | 0.011 | 2.81 |  | 0.013 | 3.27 |
| QC M | 4.04 |  | 0.096 | 2.37 |  | 0.128 | 3.16 |
| QC H | 16.16 |  | 0.301 | 1.86 |  | 0.371 | 2.29 |
| Serum L | 1.06 |  | 0.023 | 2.12 |  | 0.023 | 2.14 |
| Serum H | 15.09 |  | 0.213 | 1.41 |  | 0.345 | 2.29 |

^a^ S/Co, signal-to-cutoff ratio.
