# Supplementary material for: A new double-antigen sandwich test based on the light-initiated chemiluminescent assay for detecting anti-hepatitis C virus antibodies with high sensitivity and specificity
Source: Front Cell Infect Microbiol. 2023 Nov 24;13:1222778. doi: 10.3389/fcimb.2023.1222778 (PMC10704264; doi:10.3389/fcimb.2023.1222778)
Supplement: Supplementary file 2 [file Table_2.docx]

**Supplemental Table 2:** Evaluation of plasma equivalency to serum for the LiCA^®^ anti-HCV assay.

| Sample matrix | n |  | Negative samples^a^ | |  | Positive samples^b^ | |
| --- | --- | --- | --- | --- | --- | --- | --- |
|  |  |  | S/Co mean (SD) | Bias vs. serum mean (SD) | S/Co mean (SD) | | %Bias vs. serum mean (SD) |
| Serum | 25 |  | 0.05 (0.00) | - | 6.00 (0.44) | | - |
| Na-Citrate plasma | 25 |  | 0.05 (0.01) | 0.00 (0.01) | 5.80 (0.40) | | -3.15 (5.27) |
| K2-EDTA^c^ plasma | 25 |  | 0.05 (0.00) | 0.00 (0.01) | 5.47 (0.42) | | -8.68 (5.86) |
| K3-EDTA plasma | 25 |  | 0.05 (0.01) | 0.00 (0.01) | 5.51 (0.39) | | -7.82 (6.03) |
| Li-Heparin plasma | 25 |  | 0.05 (0.00) | 0.00 (0.01) | 5.31 (0.40) | | -11.26 (4.84) |
| Na-Heparin plasma | 25 |  | 0.05 (0.00) | 0.00 (0.01) | 5.31 (0.36) | | -11.15 (6.18) |
| CPD^c^ plasma | 25 |  | 0.06 (0.01) | 0.01 (0.01) | 5.66 (0.33) | | -5.41 (4.68) |
| CPDA^c^ plasma | 25 |  | 0.05 (0.00) | 0.00 (0.01) | 5.68 (0.35) | | -5.06 (5.91) |
| ACD-B^c^ plasma | 25 |  | 0.05 (0.01) | 0.00 (0.01) | 5.80 (0.39) | | -3.18 (5.27) |
| K-Oxalate plasma | 25 |  | 0.05 (0.01) | 0.00 (0.01) | 5.78 (0.42) | | -3.39 (6.25) |

^a^ Signal-to-cutoff (S/Co) ratios were recorded in 25 groups of HCV-free specimens. Each group were collected from the same individual and included 10 different types of matrix. Measurement with a S/Co ≥1.0 was regarded to be reactive and a negative result was considered as S/Co <1.0 for both LiCA^®^ and Architect^®^ assays.

^b^ Positive samples were prepared with the negative ones by spiking a high-positive anti-HCV specimen at 20:1 volume ratio.

^c^ EDTA, ethylenediaminetetraacetic acid; CPD, citrate phosphate dextrose; CPDA, citrate phosphate dextrose adenine; ACD-B, acid citrate dextrose solution B.
