# Supplementary material for: A new double-antigen sandwich test based on the light-initiated chemiluminescent assay for detecting anti-hepatitis C virus antibodies with high sensitivity and specificity
Source: Front Cell Infect Microbiol. 2023 Nov 24;13:1222778. doi: 10.3389/fcimb.2023.1222778 (PMC10704264; doi:10.3389/fcimb.2023.1222778)
Supplement: Supplementary file 3 [file Table_3.docx]

**Supplemental Table 3:** Detection of genotypes.

| HCV  genotype | Sample  matrix | Reactive samples/total | |
| --- | --- | --- | --- |
|  |  | LiCA^®^ anti-HCV | Architect^®^ anti-HCV |
| Genotype 1 |  | 70/70 | 70/70 |
| Genotype 1a | Serum, Citrate, CPD | 25/25 | 25/25 |
| Genotype 1b | Serum, Citrate, CPD | 26/26 | 26/26 |
| Undefined | Serum, unknown | 19/19 | 19/19 |
| Genotype 2 |  | 36/36 | 36/36 |
| Genotype 2a | EDTA, CPD | 4/4 | 4/4 |
| Genotype 2b | Citrate, CPD | 6/6 | 6/6 |
| Genotype 2c | CPD, unknown | 8/8 | 8/8 |
| Genotype 2i | CPD, unknown | 2/2 | 2/2 |
| Genotype 2k | CPD, unknown | 2/2 | 2/2 |
| Genotype 2l | CPD, unknown | 2/2 | 2/2 |
| Undefined | CPD, unknown | 12/12 | 12/12 |
| Genotype 3 |  | 41/41 | 41/41 |
| Genotype 3a | CPD, unknown | 39/39 | 39/39 |
| Undefined | Unknown | 2/2 | 2/2 |
| Genotype 4 |  | 40/40 | 40/40 |
| Genotype 4a | CPD | 11/11 | 11/11 |
| Genotype 4b | CPD | 2/2 | 2/2 |
| Genotype 4d | CPD | 8/8 | 8/8 |
| Genotype 4f | Serum, EDTA, CPD | 8/8 | 8/8 |
| Undefined | CPD, unknown | 11/11 | 11/11 |
| Genotype 5 | Heparin, CPD | 26/26 | 26/26 |
| Genotype 6 | Heparin, CPD | 26/26 | 26/26 |
| Total |  | 239/239 | 239/239 |
| S/Co^a^ mean (range) | | 149.36 (19.14~328.94) | 18.49 (10.32~28.81) |

^a^ Measurement with a ratio of signal-to-cutoff (S/Co) ≥1.0 was regarded to be reactive and a negative result was considered as S/Co <1.0 for both LiCA^®^ and Architect^®^ assays.
