# Supplementary material for: A new double-antigen sandwich test based on the light-initiated chemiluminescent assay for detecting anti-hepatitis C virus antibodies with high sensitivity and specificity
Source: Front Cell Infect Microbiol. 2023 Nov 24;13:1222778. doi: 10.3389/fcimb.2023.1222778 (PMC10704264; doi:10.3389/fcimb.2023.1222778)
Supplement: Supplementary file 4 [file Table_4.docx]

**Supplemental Table 4:** Evaluation of cross reactivity from different sources of potential interferents.

| Source of  interferents | n | Sample matrix | S/Co^a^ on LiCA^®^ anti-HCV | |  | S/Co^a^ on Architect^®^ anti-HCV | |
| --- | --- | --- | --- | --- | --- | --- | --- |
|  |  |  | Mean (SD) | Range |  | Mean (SD) | Range |
| HBsAg | 3 | Serum, Citrate | 0.07 (0.03) | 0.05~0.10 |  | 0.26 (0.32) | 0.07~0.63 |
| Anti-HAV | 3 | Citrate plasma | 0.07 (0.01) | 0.06~0.08 |  | 0.09 (0.02) | 0.07~0.10 |
| Anti-HEV | 3 | Serum, Citrate | 0.06 (0.01) | 0.05~0.06 |  | 0.11 (0.04) | 0.07~0.14 |
| Anti-HIV 1/2 | 3 | Serum, EDTA | 0.06 (0.01) | 0.05~0.06 |  | 0.14 (0.08) | 0.09~0.23 |
| Anti-Syphilis | 3 | Serum, Citrate | 0.06 (0.01) | 0.05~0.06 |  | 0.09 (0.05) | 0.05~0.14 |
| Anti-HSV 1/2 | 2 | Serum, Citrate | 0.06 (0.00) | 0.06~0.06 |  | 0.10 (0.03) | 0.08~0.12 |
| Anti-HTLV I/II | 3 | CPD plasma | 0.06 (0.01) | 0.05~0.07 |  | 0.07 (0.01) | 0.06~0.08 |
| Anti-CMV | 3 | Citrate plasma | 0.06 (0.01) | 0.06~0.07 |  | 0.06 (0.03) | 0.04~0.09 |
| Anti-EBV | 3 | Citrate plasma | 0.06 (0.01) | 0.06~0.07 |  | 0.06 (0.02) | 0.05~0.08 |
| Anti-VZV | 2 | Serum, Citrate | 0.06 (0.01) | 0.05~0.07 |  | 0.08 (0.01) | 0.07~0.08 |
| Influenza | 3 | EDTA plasma | 0.05 (0.01) | 0.05~0.06 |  | 0.07 (0.01) | 0.06~0.08 |
| HAMA | 3 | Serum | 0.09 (0.05) | 0.06~0.15 |  | 0.22 (0.07) | 0.14~0.26 |
| Hyper IgG/IgM | 3 | Serum | 0.06 (0.00) | 0.06~0.06 |  | 0.11 (0.07) | 0.05~0.19 |
| Rheumatoid factor | 3 | Serum | 0.06 (0.01) | 0.05~0.06 |  | 0.08 (0.01) | 0.07~0.09 |
| Auto-antibodies | 56 | Serum | 0.05 (0.01) | 0.04~0.07 |  | 0.09 (0.03) | 0.04~0.18 |
| SARS-CoV-2 vaccinees | 3 | Citrate plasma | 0.06 (0.00) | 0.06~0.06 |  | 0.07 (0.04) | 0.05~0.14 |
| Renal dialysis | 3 | Heparin plasma | 0.06 (0.00) | 0.06~0.06 |  | 0.08 (0.03) | 0.06~0.11 |
| Pregnant women | 3 | Serum | 0.05 (0.01) | 0.05~0.06 |  | 0.08 (0.02) | 0.06~0.09 |
| Multipara women | 3 | Serum | 0.05 (0.01) | 0.05~0.06 |  | 0.09 (0.02) | 0.08~0.11 |
| Total nonreactive samples/all tested | | | 108/108 | |  | 108/108 | |

^a^ Measurement with a ratio of signal-to-cutoff (S/Co) ≥1.0 was regarded to be reactive and a negative result was considered as S/Co <1.0 for both LiCA^®^ and Architect^®^ assays.
