# Supplementary material for: A new double-antigen sandwich test based on the light-initiated chemiluminescent assay for detecting anti-hepatitis C virus antibodies with high sensitivity and specificity
Source: Front Cell Infect Microbiol. 2023 Nov 24;13:1222778. doi: 10.3389/fcimb.2023.1222778 (PMC10704264; doi:10.3389/fcimb.2023.1222778)
Supplement: Supplementary file 5 [file Table_5.docx]

**Supplemental Table 5:** Discrepancy between LiCA^®^ and Architect^®^ anti-HCV assays in a cohort of 16,305 patient sera (n=152).

| No. (%) of samples | LiCA^®^ anti-HCV | Architect^®^ anti-HCV | Agreement Cobas^®^ vs. LiCA^®^ | RIBA 3.0 | HCV RNA | Anti-HCV confirmatory |
| --- | --- | --- | --- | --- | --- | --- |
| 4 (2.63%) | Nonreactive | Reactive | 100.00% | Positive | Negative | Positive |
| 4 (2.63%) | Reactive | Nonreactive | 100.00% | Positive | Negative | Positive |
| 1 (0.66%) | Reactive | Nonreactive | 100.00% | Indeterminate | Positive | Positive |
| 1 (0.66%) | Reactive | Nonreactive | 100.00% | Indeterminate | Negative | Positive |
|  |  |  |  |  |  |  |
| 87 (57.24%) | Nonreactive | Reactive | 98.85% | Negative | Negative | Negative |
| 46 (30.26%) | Nonreactive | Reactive | 100.00% | Indeterminate | Negative | Negative |
| 9 (5.92%) | Reactive | Nonreactive | 11.11% | Negative | Negative | Negative |
