# Supplementary material for: A new double-antigen sandwich test based on the light-initiated chemiluminescent assay for detecting anti-hepatitis C virus antibodies with high sensitivity and specificity
Source: Front Cell Infect Microbiol. 2023 Nov 24;13:1222778. doi: 10.3389/fcimb.2023.1222778 (PMC10704264; doi:10.3389/fcimb.2023.1222778)
Supplement: Supplementary file 6 [file Table_6.docx]

**Supplemental Table 6:** Subjects with RIBA-indeterminate and RNA-negative in a cohort of 16,305 patient sera (n=47).

| Architect^®^ S/Co^a^ | LiCA^®^  S/Co^a^ | Cobas^®^  S/Co^a^ | Positive bands  on RIBA |  | Architect^®^ S/Co^a^ | LiCA^®^  S/Co^a^ | Cobas^®^  S/Co^a^ | Positive bands  on RIBA |
| --- | --- | --- | --- | --- | --- | --- | --- | --- |
| 0.94 | 54.73 | 59.66 | NS3, NS4 |  | 2.08 | 0.16 | 0.04 | NS3, Helicase |
| 1.08 | 0.12 | 0.03 | NS3, Helicase |  | 2.15 | 0.07 | 0.04 | NS3, Helicase |
| 1.08 | 0.08 | 0.03 | NS3, NS4 |  | 2.18 | 0.06 | 0.05 | NS3, NS4 |
| 1.13 | 0.13 | 0.03 | NS3, Helicase |  | 2.25 | 0.12 | 0.03 | NS3, Helicase |
| 1.16 | 0.04 | 0.04 | NS3, Helicase |  | 2.34 | 0.16 | 0.04 | NS3, Helicase |
| 1.22 | 0.09 | 0.04 | NS3, NS5 |  | 2.51 | 0.05 | 0.13 | NS3, Helicase |
| 1.28 | 0.12 | 0.06 | Helicase |  | 2.54 | 0.07 | 0.04 | NS3, Helicase |
| 1.28 | 0.14 | 0.06 | NS3, NS4 |  | 2.68 | 0.13 | 0.17 | NS3, Helicase |
| 1.32 | 0.08 | 0.06 | Helicase |  | 2.73 | 0.05 | 0.04 | Core1 |
| 1.38 | 0.10 | 0.04 | Core1 |  | 2.86 | 0.07 | 0.03 | NS3, Helicase |
| 1.47 | 0.11 | 0.04 | NS4, Helicase |  | 2.94 | 0.14 | 0.04 | NS3, Helicase |
| 1.49 | 0.07 | 0.04 | NS3, Helicase |  | 3.25 | 0.08 | 0.07 | NS3, Helicase |
| 1.52 | 0.12 | 0.04 | NS3, Helicase |  | 3.42 | 0.06 | 0.04 | NS3, Helicase |
| 1.52 | 0.11 | 0.04 | NS3, Helicase |  | 3.52 | 0.05 | 0.06 | NS3, Helicase |
| 1.53 | 0.10 | 0.04 | NS3, Helicase |  | 4.50 | 0.17 | 0.03 | NS3, Helicase |
| 1.55 | 0.08 | 0.05 | NS3, NS4 |  | 4.57 | 0.09 | 0.04 | NS3, Helicase |
| 1.57 | 0.09 | 0.04 | NS3, Helicase |  | 4.76 | 0.07 | 0.04 | Core1 |
| 1.58 | 0.04 | 0.04 | NS3, Helicase |  | 5.11 | 0.08 | 0.15 | Core1 |
| 1.70 | 0.05 | 0.04 | NS3, Helicase |  | 5.80 | 0.13 | 0.03 | NS3, Helicase |
| 1.74 | 0.08 | 0.07 | NS3, Helicase |  | 7.11 | 0.07 | 0.04 | NS4, Helicase |
| 1.78 | 0.12 | 0.05 | NS3, Helicase |  | 8.33 | 0.14 | 0.03 | NS3, Helicase |
| 1.85 | 0.09 | 0.04 | NS5, Helicase |  | 9.77 | 0.10 | 0.04 | NS3, Helicase |
| 1.85 | 0.12 | 0.04 | Core1 |  | 11.07 | 0.07 | 0.04 | NS3, NS4 |
| 1.93 | 0.08 | 0.04 | NS3, Helicase |  |  |  |  |  |

^a^ Measurement with a ratio of signal-to-cutoff (S/Co) ≥1.0 was regarded to be reactive and a negative result was considered as S/Co <1.0 for both LiCA^®^ and Architect^®^ assays. For the Cobas^®^ assay, S/Co ≥1.0 was reactive and S/Co <0.9 was non-reactive, while S/Co between 0.9 and 1.0 was classified to be borderline.
