# Supplementary material for: A new double-antigen sandwich test based on the light-initiated chemiluminescent assay for detecting anti-hepatitis C virus antibodies with high sensitivity and specificity
Source: Front Cell Infect Microbiol. 2023 Nov 24;13:1222778. doi: 10.3389/fcimb.2023.1222778 (PMC10704264; doi:10.3389/fcimb.2023.1222778)
Supplement: Supplementary file 7 [file Image_1.pdf]

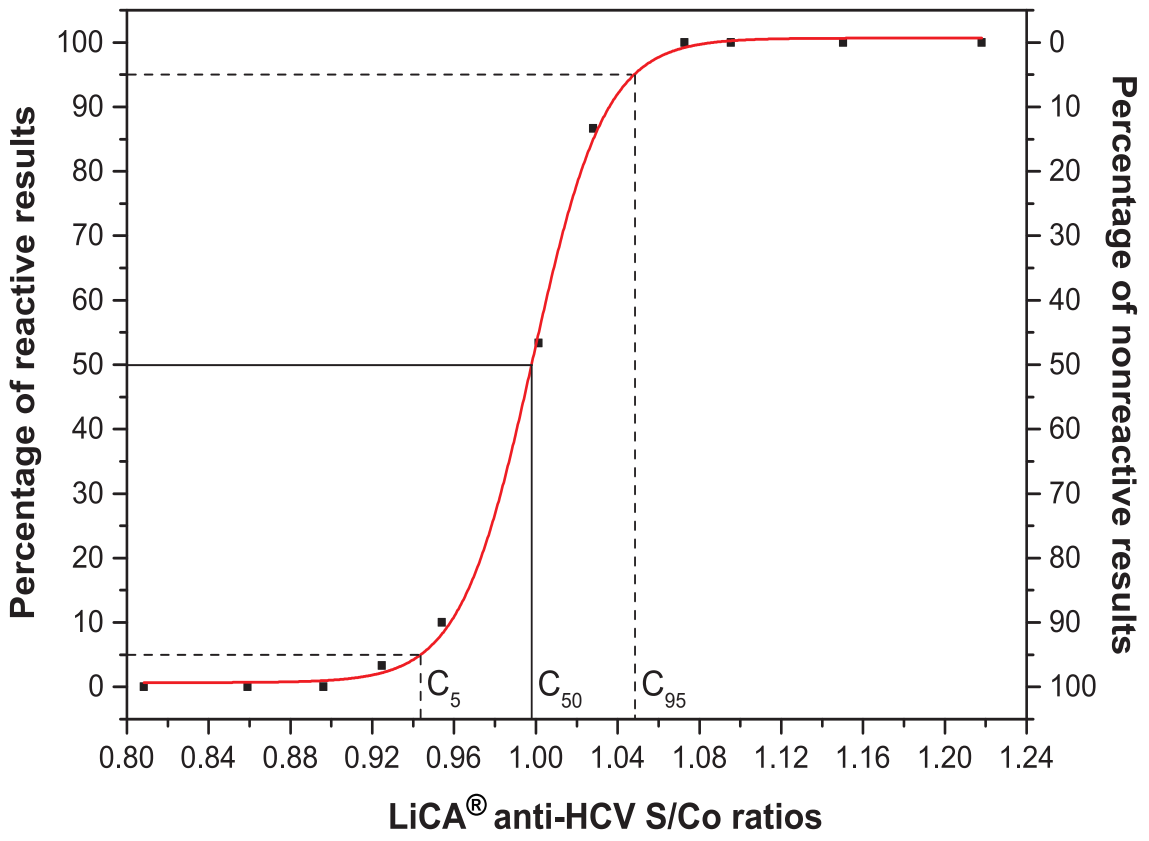

Supplementary Figure 1. C50 imprecision analysis for the LiCA® anti-HCV assay in serum following the EP17-A2 protocol.

The C5~C95 interval was calculated to be -5.44%~5.03% away from C50.
