# Supplementary material for: A new double-antigen sandwich test based on the light-initiated chemiluminescent assay for detecting anti-hepatitis C virus antibodies with high sensitivity and specificity
Source: Front Cell Infect Microbiol. 2023 Nov 24;13:1222778. doi: 10.3389/fcimb.2023.1222778 (PMC10704264; doi:10.3389/fcimb.2023.1222778)
Supplement: Supplementary file 8 [file Image_2.pdf]

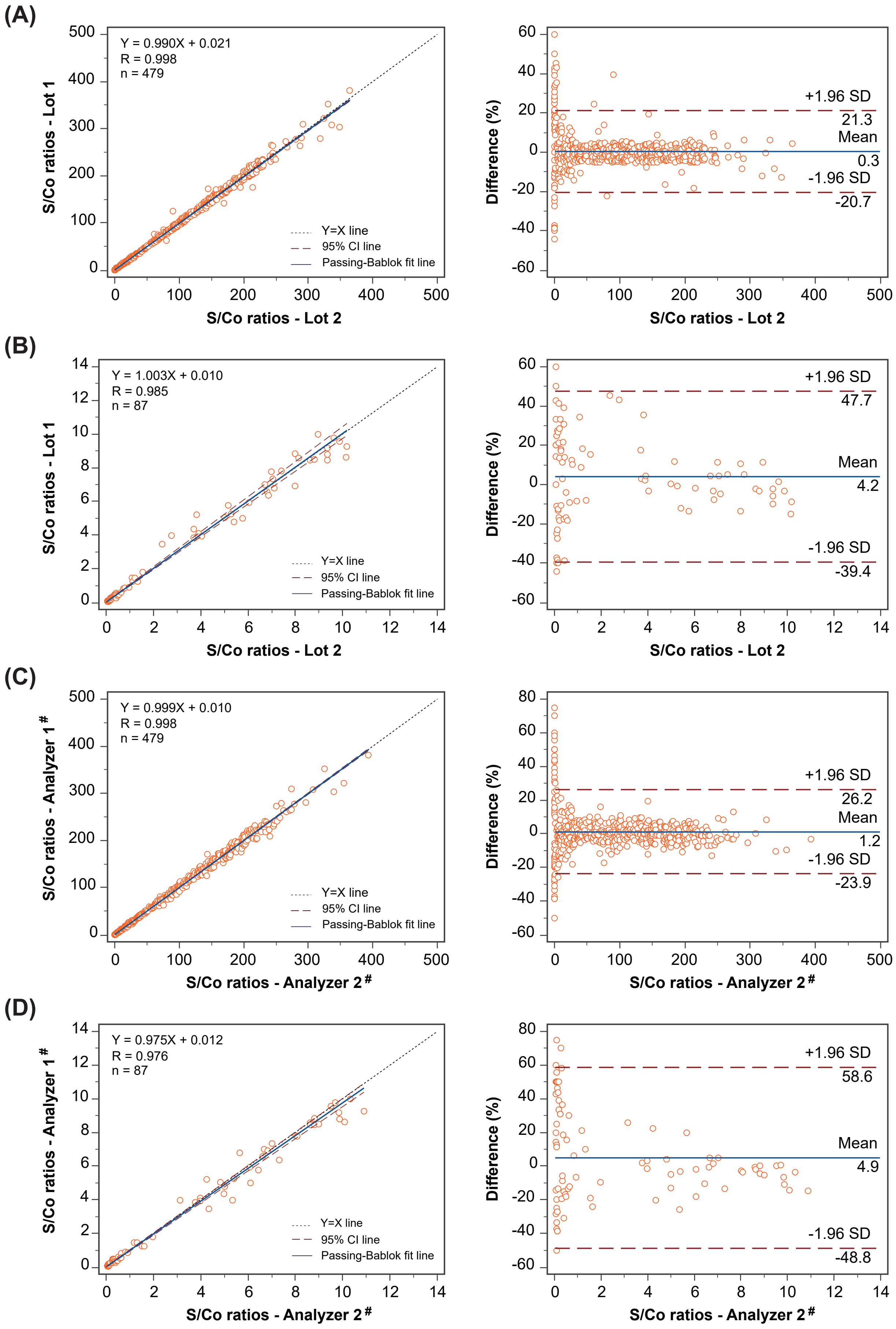

Supplementary Figure 2. Passing-Bablok regression and Bland-Altman plot for evaluation of variability in lot-to-lot reagents and between-instruments following the EP09c protocol.

(A)and (C) were plotted using serum samples with a broad range of signal-to-cutoff (S/Co) ratios from 0.05~380, while (B) and (D) were analyzed with a low range of S/Co (<10) samples.
