# Supplementary material for: A new double-antigen sandwich test based on the light-initiated chemiluminescent assay for detecting anti-hepatitis C virus antibodies with high sensitivity and specificity
Source: Front Cell Infect Microbiol. 2023 Nov 24;13:1222778. doi: 10.3389/fcimb.2023.1222778 (PMC10704264; doi:10.3389/fcimb.2023.1222778)
Supplement: Supplementary file 9 [file Image_3.pdf]

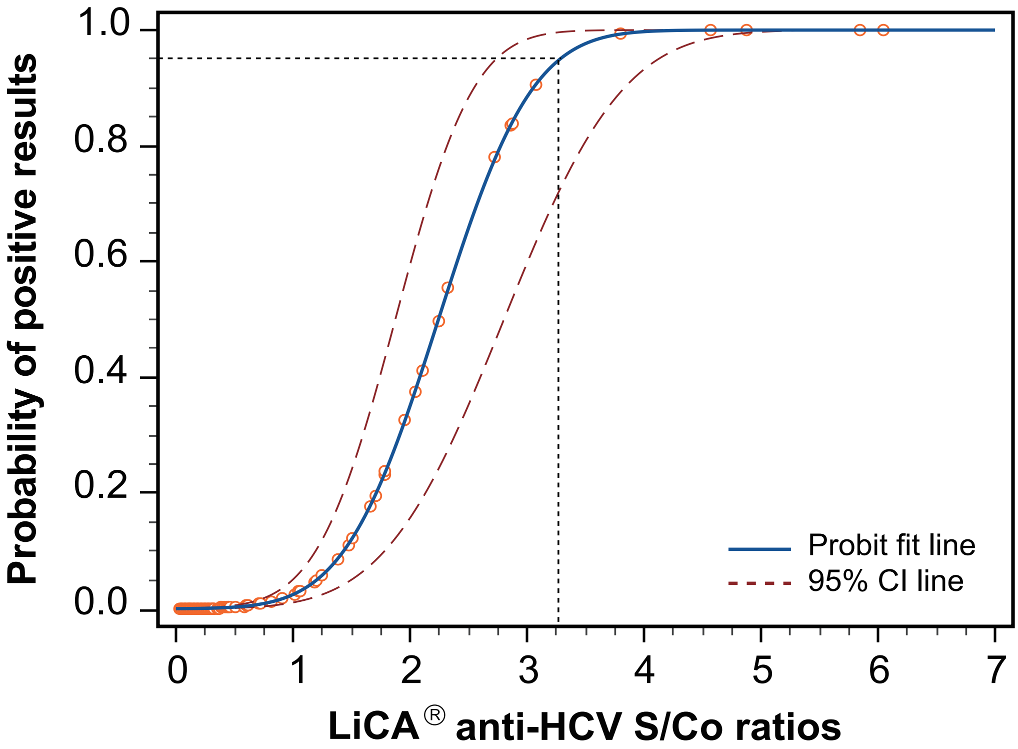

Supplementary Figure 3. Probit regression for identifying the signal-to-cutoff (S/Co) ratio in prediction of a positivity ≥95%.

Probit regression was performed using clinical patient serum samples (n=16,305). The predicting S/Co value with a positivity ≥95% was calculated to be 3.28 (95% CI, 2.74~4.12) for the assay.
