# Supplementary material for: A new double-antigen sandwich test based on the light-initiated chemiluminescent assay for detecting anti-hepatitis C virus antibodies with high sensitivity and specificity
Source: Front Cell Infect Microbiol. 2023 Nov 24;13:1222778. doi: 10.3389/fcimb.2023.1222778 (PMC10704264; doi:10.3389/fcimb.2023.1222778)
Supplement: Supplementary file 10 [file Image_4.pdf]

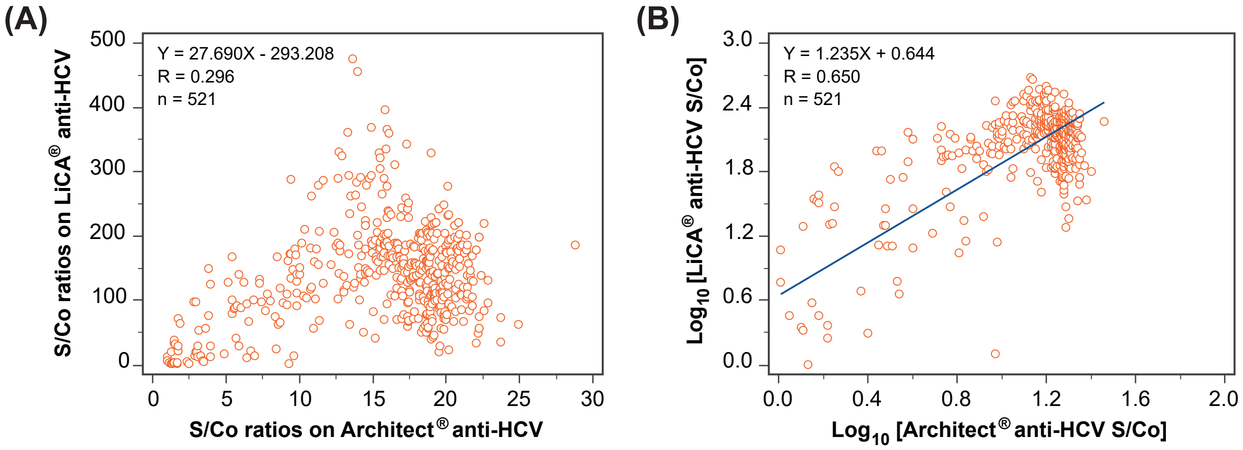

Supplementary Figure 4. Correlation of signal-to-cutoff (S/Co) ratios between LiCA® and Architect® anti-HCV assays in clinical patient serum samples with S/Co ⩾1.0.

(A) Deming regression for S/Co ratios. (B) Deming regression for Log10 [S/Co].
